# Why lithium studies for ALS treatment should not be halted prematurely

**DOI:** 10.3389/fnins.2014.00267

**Published:** 2014-09-02

**Authors:** Galila Agam, Adrian Israelson

**Affiliations:** ^1^Department of Clinical Biochemistry and Pharmacology, Faculty of Health Sciences and Psychiatry Research Unit, Ben Gurion University of the NegevBeer Sheva, Israel; ^2^Department of Physiology and Cell Biology, Faculty of Health Sciences, Ben Gurion University of the NegevBeer Sheva, Israel

**Keywords:** lithium, ALS, effective blood levels, riluzole, mouse strains

Perrin's recent comment (Perrin, 2014) emphasizes that mouse studies must be sibling-matched, gender-balanced, and investigator-blinded. To substantiate his claim, Perrin provides examples from studies on lithium as a treatment for ALS, describing them as misleading. While we fully agree that there is a need for better designed preclinical studies and better characterization of disease models, Perrin's examples are problematic. First, Perrin's pharmacokinetic calculations of the dosing regimens predict plasma lithium levels of 0.3–1.68 meq/L. However, given that the beneficial therapeutic window in bipolar-disorder is 0.6–1.5 meq lithium/L, it is conceivable that only regimens resulting in blood levels ≥0.6 meq/L would lead to beneficial results. Second, instead of theoretical calculations lithium levels should be measured directly. We recently reported that lithium blood levels of ICR mice bred separately in the USA and Israel, but treated in the same facility with the same supplemented food, differed by 2.5-fold (Sade et al., 2014). This difference may explain why, despite using the same regime, the Fornai et al. (2008) and Gill et al. (2009) studies produced opposite results. Third, daily intraperitoneal lithium injections rather than the less irritating food supplementation method might also contribute to unsuccessful outcomes.

In negative ALS clinical trials (UKMND-LiCALS Study Group et al., 2013) plasma lithium levels were 0.4–0.8 meq/L, barely at the lower therapeutic range for psychiatric disorders and significantly lower than the Ki for lithium of its major hypothesized targets (≥1 mM). Indeed, in the antidepressant-like rodent model, the forced-swim test, only plasma lithium levels above 1.3 meq/L significantly reduced immobility-time (Bersudsky et al., 2007). Importantly, it has recently been shown (Yáñez et al., 2014) that lithium-induced neuroprotection is antagonized by riluzole (the only FDA-approved drug for ALS), suggesting that the drug's neurotoxic effects may mask the potential neuroprotective activity of lithium.

In conclusion, we believe that the potential beneficial effect of lithium for neurodegenerative disorders deserves serious reconsideration.

## Conflict of interest statement

The authors declare that the research was conducted in the absence of any commercial or financial relationships that could be construed as a potential conflict of interest.
